# A Cross-Sectional Study to Correlate Serum Complement C3 and C4 Levels With Clinical and Pathological Severity in Cutaneous Small-Vessel Vasculitis

**DOI:** 10.7759/cureus.24845

**Published:** 2022-05-09

**Authors:** Namrata Sarkar, Aparna Palit, Madhusmita Sethy, Biswanath Behera, Siddhartha Dash, Dinesh P Sahu

**Affiliations:** 1 Dermatology and Venereology, All India Institute of Medical Sciences, Bhubaneswar, Bhubaneswar, IND; 2 Dermatology and Venereology, All India Institute of Medical Sciences, Kalyani, Kalyani, IND; 3 Pathology and Laboratory Medicine, All India Institute of Medical Sciences, Bhubaneswar, Bhubaneswar, IND; 4 Community Medicine, All India Institute of Medical Sciences, Bhubaneswar, Bhubaneswar, IND

**Keywords:** vasculitis, vascular, skin diseases, fluorescent antibody technique, complement

## Abstract

Introduction

The role of serum C3 and C4 levels as a marker of disease activity in cutaneous small-vessel vasculitis (CSVV) has been sparsely studied, especially in India. The primary objective was to determine the correlation between clinico-histopathological severity and serum C3 and C4 levels in CSVV. The secondary objective was to determine the association between direct immunofluorescence (DIF) findings and serum C3 and C4 levels and clinico-histopathological findings.

Method

This prospective cross-sectional study included all the clinically diagnosed cases of CSVV that satisfied the pathological criteria for CSVV. A clinical disease activity grade and a histopathological severity grade were calculated in all patients (N=50).

Results

Serum C3 and C4 levels (n=44) were diminished in 4.5% of cases. There was no significant correlation between the serum C3 and C4 levels and the clinical and histopathological severity. DIF was positive in 60.0% of cases (n=45), and IgA was the predominant immune deposit (46.7%). No significant association was detected between the DIF findings and the serum C3 and C4 levels, histopathological severity, and clinical disease activity grade. Positive DIF findings were significantly associated with palpable purpura and cutaneous necrosis. A significant association was detected between gastrointestinal involvement and IgA positivity.

Conclusion

In CSVV, serum C3 and C4 may not be used as markers of disease severity, and a positive DIF finding may indicate an underlying gastrointestinal involvement.

## Introduction

Cutaneous small-vessel vasculitis (CSVV) causes inflammation and destruction of primarily postcapillary venules and the capillaries [1]. It can have varied morphological presentations; however, the common cutaneous manifestations include crops of symmetric palpable purpura, purpuric macules, and partially blanchable urticarial papules and plaques. The formation of circulating immune complexes due to multiple possible triggers, and their deposition in vessels, followed by a local and possibly, systemic complement activation and resulting inflammation, is the most accepted pathomechanism of CSVV. The positive direct immunofluorescence (DIF) findings in CSVV support this theory. The circulating immune complexes get lodged within small vessels in the superficial dermis, joints, gastrointestinal tract, or glomeruli. An associated extracutaneous involvement is noted in 20 to 50% of the cases; renal and musculoskeletal systems are commonly affected [1-5].

Histopathological examination remains the gold standard for the diagnosis of CSVV. The prognosis of CSVV depends on the severity of the systemic involvement [6]. The underlying systemic involvement may be difficult to identify in the absence of specific systemic symptoms clinically. There are conflicting reports on the association or correlation between clinical and histopathological severity [5,7,8]. The complement C3 and C4 levels are done in CSVV to rule out associated systemic vasculitis and low complement levels suggest immune-complex consumption and systemic involvement [9].

The prognostic role of serum C3 and C4 levels as markers of disease activity in CSVV has been sparsely studied, especially in India. The primary objective was to determine the correlation between the severity of clinico-histopathological findings and serum complement C3 and C4 levels in CSVV. The secondary objective was to determine the association between DIF findings and serum complement C3 and C4 levels, and clinico-histopathological findings in CSVV.

## Materials and methods

Following institute ethics clearance, this prospective cross-sectional study was conducted in the outpatient department of dermatology and venereology of a tertiary care hospital in India from July 2019 to April 2021. Patients with a clinical diagnosis of CSVV, irrespective of age and gender, were included in the study after obtaining informed consent. We excluded the patients who presented with clinical features simulating CSVV (vasculitis-mimics) but lacked histopathological evidence, patients with overlapping clinical patterns of small and medium or large vessel vasculitis, and patients who did not give consent for a skin biopsy. Detailed history and clinical examination were done to elicit the underlying cause, if any, and to detect any systemic involvement. History of drug exposure, if within eight weeks of the appearance of lesions, was considered significant [2].

Disease activity grade

As proposed by Dauchel et al., a clinical disease activity grade was used to assess the disease activity in each patient [10]. The following three parameters were used: (i) clinical extent of the lesions (1=lesions were distributed below the waist, 2= lesions were extending above the waist); (ii) evidence of cutaneous necrosis (0= absent, 1= present); and (iii) clinical evidence of systemic involvement (scored 1 if any one of the following general features or evidence of organ involvement was present: fever, myalgia, arthralgia, arthritis, renal involvement, pulmonary involvement and scored 2 if more than one of these features were present). Patients with scores 1-2 were considered to have grade I disease activity, score 3 as grade II, and score 4 or more as grade III.

In all patients, baseline investigations including complete hemogram, erythrocyte sedimentation rate (ESR), serum urea and creatinine, liver function test, hepatitis screen, antinuclear antibody (ANA), anti-neutrophil cytoplasmic antibody (ANCA), stool for occult blood and urine microscopy was done. In addition, serum C3 (reference range: 75.0 - 135.0 mg/dL) and C4 levels (reference range: 9.0-36.0 mg/dL) were done for all patients using the turbidimetric immunoassay test. A value less than the lower limit of the reference range was considered as diminished.

Two skin biopsy samples (using a 4-mm punch), one for histopathological examination and one for the DIF test, were taken from the latest and most proximal representative skin lesions. Histopathological evaluation was done by two authors independently. The presence of two or more of the following pathological features were considered to be confirmatory of the histopathological diagnosis of CSVV: Small vessel wall disruption, Fibrin deposition within the lumen and, or vessel wall, the presence of nuclear dust [11]. A semiquantitative grading was used to assess the severity of the histopathological findings in each case (Table 1). The total score ranged from 0 to 20 and was subdivided into three grades (Grade I/mild = 0 to 7, Grade II/moderate= 8 to 14, and Grade III/severe= 15 to 20).

**Table 1:** Histopathological grading criteria

Parameters	Score (1=mild, 2= moderate, 3=severe)
Vessel wall damage	Absent = 0
	<25% of the circumference involved = 1
	25-50% of the circumference involved = 2
	>50% of the circumference involved = 3
Fibrin deposition on the vessel wall	Absent = 0
	<25% of the circumference involved = 1
	25-50% of the circumference involved = 2
	>50% of the circumference involved = 3
Red blood cell extravasation	Absent = 0
	Present in <25% area per high power field (HPF) = 1
	Present in 25-50% area per HPF = 2
	>50% area per HPF = 3
Perivascular edema	Absent = 0
	Mild = 1
	Moderate = 2
	Perivascular severe edema ± interstitial edema = 3
Depth of the infiltrate	Involve only the superficial dermal plexus = 1
	Involve superficial + deep dermal plexus = 2
	Involve superficial and deep dermal plexus and subcutaneous vessels=3
Inflammatory infiltrate	Absent = 0
	Present in <25% area per HPF = 1
	Present in 25-50% area per HPF = 2
	Present in >50% area per HPF = 3
Number of blood vessel involved (of the whole section)	Absent = 0
	<25% of the blood vessels = 1
	25-50% of the blood vessels = 2
	>50% of the blood vessels = 3

The sample for the DIF study was sent in the phosphate-buffered saline (PBS) medium. Antibody- fluorophore (FITC) tagged conjugates to IgG, IgM, IgA, and C3 were used. The location and the immunofluorescence pattern for each immune deposit were observed using the fluorescent microscope employing a FITC filter, and photographs were captured using the GenAsis software. The intensity of each immunofluorescence was graded as 0, 1+, 2+, and 3+.

The sample size was calculated (N = [(Zα+Zβ)/C]2 + 3) to be 47, considering an expected correlation coefficient (r) = 0.400 [12].

Statistical analysis

The statistical analysis of data was done using IBM Corp. Released 2011. IBM SPSS Statistics for Windows, Version 20.0. Armonk, NY: IBM Corp. A test of normality was done using the Shapiro-Wilk test. The data set of serum C3 and C4 values were found to be nonparametric. Categorical variables were presented as percentages or proportions. Continuous variables were presented as mean with standard deviation or median with range. Test of significance for continuous variables was done using Kruskal Wallis test and Mann Whitney U test, as applicable. The Chi-square test and Fisher’s Exact test were used to find the association between the different categorical data. Correlation analysis was done using the Spearman rho test for nonparametric continuous data and Kendall’s tau-b test for ordinal data. For all purposes, P-value <0.05 was considered significant.

## Results

**Figure 1:**
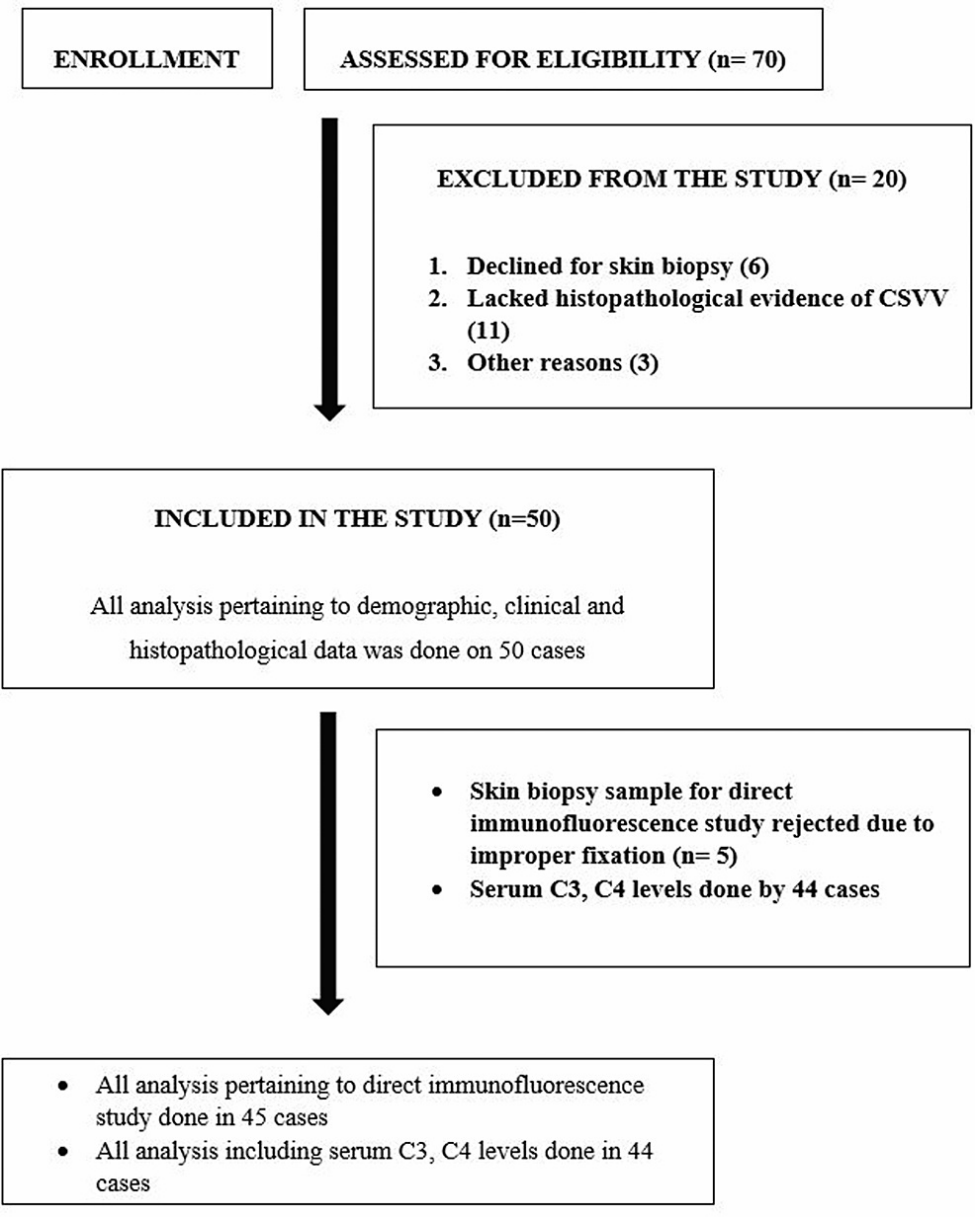
Study participant’s flow chart Figure 1: Participant's flow chart

A total of 50 patients of CSVV were included for the analysis in this study. The participants’ flow chart is mentioned in Figure 1. Table 2 shows the clinical and demographic characteristics and laboratory abnormalities in our cases.

**Table 2:** Demographic and clinical profile of the included cases (N=50)

Variable				n (%)
Male				24 (52.0)
Female				26 (48.0)
Age (years), mean ± standard deviation (range)				33.6±16.8 (2-78)
Disease duration (days), median (range)				75 (2-5475)
First episode				26 (52.0)
One or more episodes in the past				24 (48.0)
Symptoms	Itching			26 (52.0)
	Pain at lesional site/ burning sensation			16 (32.0)
	Fever			15 (30.0)
	Myalgia			5 (10.0)
	Arthralgia			21 (42.0)
	Gastrointestinal symptoms			7 (14.0)
Distribution of the skin lesions	Lower limbs			12 (24.0)
	Lower limbs, upper limbs and, or trunk			30 (60.0)
	Generalized (including face)			8 (16.0)
Cutaneous morphology		Palpable purpura		28 (56.0)
		Purpuric macule		2 (4.0)
		Erythematous maculopapular rash		1 (2.0)
		Urticated plaque/ papule		23 (46.0)
		Annular urticated plaque		8 (16.0)
		Vesicles/ bullae		6 (12.0)
		Surface ulceration/ crusting		4 (8.0)
		Cutaneous necrosis		8 (16.0)
		Retiform purpura		4 (8.0)
		Pustule		2 (4.0)
General examination	Lymphadenopathy			3 (6.0)
	Pallor			17 (34.0)
	Edema of hand and feet			22 (44.0)
Systemic examination Arthritis (Joint swelling/ tenderness)				11 (22.0)
Laboratory abnormalities			Anemia	10 (20.0)
			Leucocytosis	5 (10.0)
			Leucopenia	8 (16.0)
			Elevated erythrocyte sedimentation rate	34 (68.0)
			Elevated C-reactive protein	6 (12.0)
			Deranged liver function test	4 (8.0)
			Elevated creatinine	3 (6.0)
			ANA positive	3 (6.0)
			Hematuria	2 (4.0)
			Glucosuria	2 (4.0)
			Proteinuria	3 (6.0)
			Occult blood in stool	4 (8.0)

**Figure 2:**
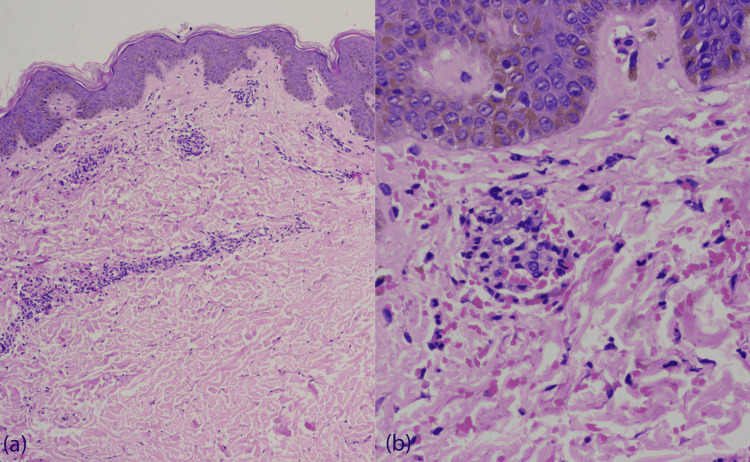
(a) Grade II histopathology grade (H & E, X100). (b) Grade II histopathology grade (H & E, X400) (a) Grade II histopathology grade- Histology shows vessel wall disruption, fibrin deposition, perivascular neutrophilic infiltration, leukocytoclasia, and erythrocyte extravasation (H & E, X100). (b) Grade II histopathology grade- Vessel wall disruption, fibrin deposition and leukocytoclasia (H & E, X400)

**Figure 3:**
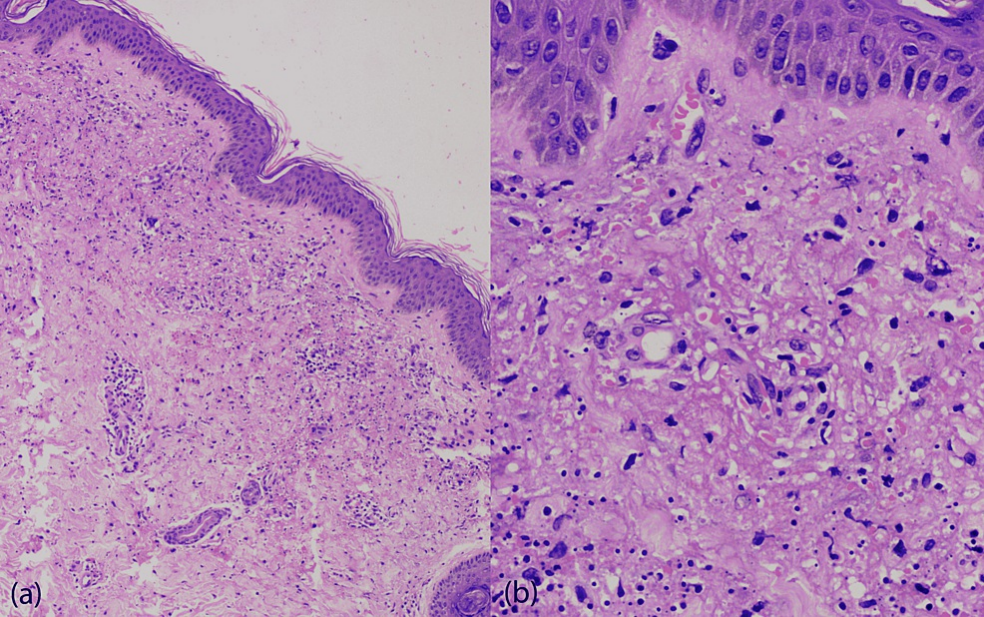
(a) Grade III histopathology grade (H & E, X100). (b) Grade III histopathology grade (H & E, X400) Figure 3: (a) Grade III histopathology grade- Histology shows vessel wall destruction, fibrin deposition on the wall and perivascular area, marked leukocytoclasia, and erythrocyte extravasation (H & E, X100). (b) Grade III histopathology grade- Extensive vessel wall disruption, fibrin deposition and leukocytoclasia (H & E, X400)

A known trigger for the current episode of CSVV was identified in 15 (30.0%) cases and included new drug intake, infection, physical exertion, exposure to cold, pregnancy, and sudden stoppage of oral steroids. Antecedent history of a new drug intake was present in 7 (14.0%) cases. Systemic involvement (history and laboratory test) was present in 17 (34.0%) cases; arthritis in 11 (22.0%), gastrointestinal involvement in 8 (16.0%), and renal involvement in 4 (8.0%) patients. Mild clinical disease activity (grade I) was seen in 29 (58.0%) cases, moderate (grade II) in 15 (30.0%) cases and severe (grade III) in 6 (12.0%) cases. Serum C3 and C4 levels were done in 44 cases. Serum C3 was diminished in two cases (4.5%, N=44), and serum C4 was diminished in two cases (4.5%, N=44). The histopathological details of all the patients are enlisted in Table 3. Of the 50 patients, moderate severity/grade II was noted in 33 (66.0%) cases and severe/grade III in 17 (34.0%) cases (Figures 2, 3). 

**Table 3:** Histopathological features observed in the study

Histopathological parameter		n (%)
Pattern of involvement	Perivascular	25 (50.0)
	Perivascular and interstitial	25 (50.0)
Type of blood vessel involved	Capillary and venule	46 (92.0)
	Capillary, venule, and arteriole	4 (8.0)
Inflammatory infiltrate	Neutrophil	44 (88.0)
	Lymphocyte	31 (62.0)
	Eosinophil	3 (6.0)
Endothelial swelling		50 (100.0)
Thrombosis		1 (2.0)
Red blood cell extravasation (percentage per high power field)	Absent	16 (32.0)
	Mild	11 (22.0)
	Moderate	9 (18.0)
	Severe	14 (28.0)
Vessel wall damage (percentage of circumference)	Absent	0 (0.0)
	Mild	19 (38.0)
	Moderate	10 (20.0)
	Severe	21 (42.0)
Fibrin deposition (percentage of vessel wall circumference involved)	Absent	5 (10.0)
	Mild	19 (38.0)
	Moderate	10 (20.0)
	Severe	16 (32.0)
Perivascular edema	Absent	1 (2.0)
	Mild	42 (84.0)
	Moderate	5 (10.0)
	Severe perivascular edema ± interstitial edema	2 (4.0)
Depth of the inflammatory infiltrate	Mild	46 (92.0)
	Moderate	4 (8.0)
	Severe	0 (0.0)
Perivascular inflammatory infiltrate (percentage per high power field)	Absent	0 (0.0)
	Mild	12 (24.0)
	Moderate	15 (30.0)
	Severe	23 (46.0)
Number of blood vessels involved (of the whole section)	Mild	0 (0.0)
	Moderate	0 (0.0)
	Severe	50 (100.0)
Nuclear dust		35 (70.0)

There was no significant correlation between the serum complement C3 and C4 levels and the clinical and histopathological severity (Table 4). There was no significant correlation between the clinical and histopathological severity in our cases (Correlation coefficient = −0.118, P-value = 0.390).

**Table 4:** Correlation between serum complement levels C3 and C4 and clinical and histopathological severity grade

Correlations (Spearman rho)		Serum C3 level (N=44)	Serum C4 level (N=44)
Clinical disease activity grade	Correlation coefficient	0.053	0.120
	P- value	0.733	0.439
Histopathological severity grade	Correlation coefficient	−0.193	0.05
	P- value	0.208	0.746

**Figure 4:**
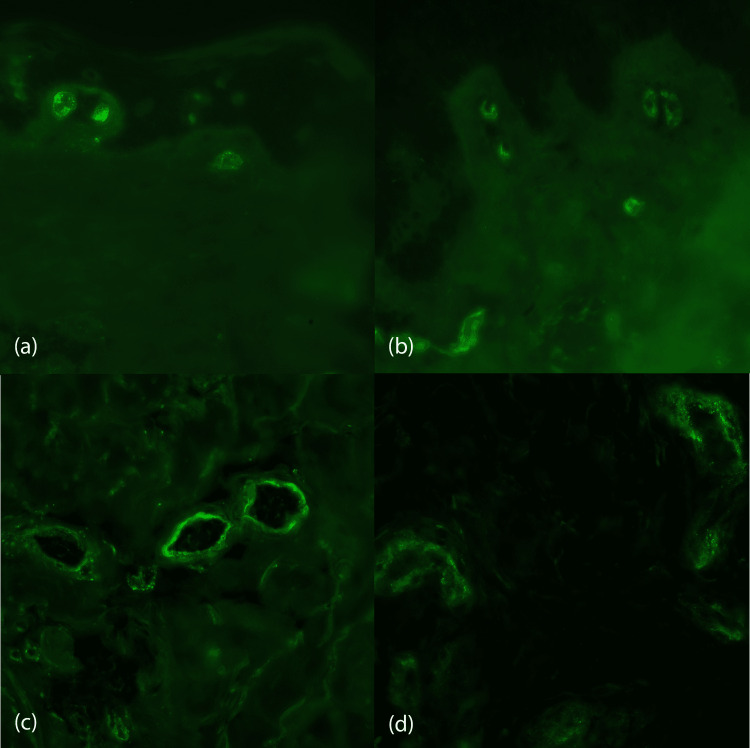
DIF shows granular deposit of (a) IgA, (b) C3, (c) IgG and (d) IgM on the vascular wall (DIF, X100, X100, X400, X400) Figure 4: Direct immunofluorescence (DIF) shows granular deposit of (a) IgA, (b) C3, (c) IgG and (d) IgM on the vascular wall in cases of cutaneous small-vessel vasculitis (DIF, X100, X100, X400, X400)

Out of the 50 included cases of CSVV, DIF could be done for 45 (90.0%) cases, whereas in five cases, the sample for DIF was rejected due to improper fixation. DIF was positive in 27 (60.0%) cases and negative in 18 (40.0%) cases. The pattern of immunofluorescence observed in all cases was granular. Of the 27 DIF-positive cases, the immune deposits were distributed along the walls of the superficial vessels in 11 (24.4%) cases and a perivascular location in 16 (35.6%) cases (Figure 4). Table 5 shows the distribution of the immune deposits in our cases according to the duration of the lesions biopsied. The predominant immune deposit in our cases was IgA (n=21, 46.7%). No statistically significant association was detected between the DIF positivity and the serum complement C3 and C4 levels, histopathological severity, and clinical disease activity grade (P-value >0.05). Palpable purpura (P-value= 0.007) and the presence of cutaneous necrosis (P-value= 0.014) had a significant association with DIF positivity, whereas urticated lesions had a negative association with positive DIF in our patients (P-value= 0.001). A significant association was detected between IgA positivity and involvement of the gastrointestinal system (P-value= 0.039).

**Table 5:** Distribution of immune-deposits in our cases according to the duration of the lesions biopsied

Age of the lesion biopsied	Total cases	IgM	IgA	IgG	C3
<48 hours	32	4	17	1	10
48-72 hours	10	0	3	0	3
>72 hours	3	1	1	1	2
Total	45	5 (10.0)	21 (42.0)	2 (4.0)	15 (30.0)

## Discussion

The prognosis of a patient with CSVV depends on systemic involvement, which leads to a great deal of morbidity and rarely mortality. Besides, the use of systemic corticosteroids and, or immunosuppressive drugs is determined by the underlying organ involvement [6]. So, it is imperative to detect systemic involvement early in the course of the disease. In this study, we tried to correlate the clinical and pathological severity in CSVV with serum C3 and C4 levels.

Our patients’ mean age at presentation was 33.6 years (range: 2-78 years). There was no gender preponderance in our study, similar to previous studies on CSVV [4]. The majority (50.0%) of the patients in our series were young adults (age group 20-39 years), as described before [5,13]. The median duration of the CSVV in this study was 75 days, ranging from 2 days to 15 years. In the index study, around one-third (30%) of the patients had exposure to a known trigger. Antibiotics were commonly implicated (cephalosporin, ciprofloxacin, azithromycin), followed by NSAIDs. A possible aetiological factor was identified in 26.0% in the current study. An underlying condition or a causal agent in the range of 20-85% of cases has been reported in previous studies [6,14-18]. The initial symptom in most of our patients was skin-related: skin lesion, itching and, or burning sensation. On clinical examination, a systemic involvement was found in 28.0% of our cases. Previous studies had reported a systemic involvement in 20% to 50% of patients [2-4]. In concordance with two prior Indian studies, arthritis followed by gastrointestinal features were the most common systemic symptoms in our series [2,5]. In this study, cutaneous vasculitis lesions were commonly located on the leg and the ankle. Palpable purpura was the most common (56.0%) morphological presentation of CSVV followed by urticated papules and plaques (46.0%) in our patients, in concordance with previous studies [4,5].

More than half of the patients in this study had a mild clinical disease activity (58.0%), and 12.0% of patients had severe clinical disease activity. In contrast, by using the same disease activity score, Dauchel et al. noticed moderate clinical disease activity in the majority of the cases (44.8%, N=29) [10]. It may be due to the early presentation of our cases, or they had received prior treatment.

Only a small fraction of our patients (4.5%) had diminished serum C3 and C4. A similar finding for serum C3 was noted by Sais et al. (6% cases) and by Bouiller et al. (7.9% cases). In contrast to our study, Sais et al. reported a diminished serum C4 level in 25% of cases, and Bouiller et al. reported a diminished serum C4 level in 16.4% of cases [3,4].

Dauchel et al. reported a good correlation between the complement activation products in plasma, C3d, g, and terminal complement components (TCC) with the clinical severity of vasculitis. However, serum C3 level was not correlated with the histopathological or clinical severity. Even though the study demonstrated the activation of the classical complement pathway and consumption of complement factors as a major mechanism in the pathophysiological evolution of the disease, it did not show a significant decrease in the serum C3 and C4 in their patients. Similarly, there was no correlation between serum C3 and C4 levels with clinical disease severity and histopathological severity in the index study. Dauchel et al. also had demonstrated a lack of correlation between the plasma levels of complement activation products (C3d, g, and TCC) and the intensity of cutaneous perivascular deposits of C3d, g, and TCC. They concluded that the increase in the plasma levels of these products was due to systemic activation of complements rather than a passive diffusion from the cutaneous deposits [10]. Our study could not find a significant association between serum C3 and C4 levels with the immunofluorescence findings. Similarly, a study done by Alalwani et al. did not find any significant association between low C3 and low C4 levels with DIF positivity [19].

We did not find a statistically significant correlation between the clinical disease activity and histopathological severity grades. A similar finding was noted by Cribier et al. and Khetan et al. They concluded that the histopathological changes of LCV could not predict the extracutaneous complications in vasculitis [5,8]. In contrast, Ratnam et al. noted a significant association between the clinical severity and histopathological severity in the cutaneous vasculitis cases that presented with palpable purpura [7].

We did not notice any significant correlation between the individual histopathological parameters and the clinical disease severity in our cases. In contrast, Hodge et al. found a statistically significant relationship between the clinical severity with the severity of individual histopathological parameters: vessel wall changes, exocytosis, depth of the inflammation, leukocytoclasia, and fibrinoid necrosis, however, their cases had high individual variation, and they had concluded that predicting clinical outcome based on histopathological findings was not reliable [20].

The details of DIF findings in various studies in CSVV are mentioned in Table 6. The deposition in our cases had a granular pattern on DIF similar to previous studies [21]. In the index study, IgA was the most frequent immune deposit (46.7%), similar to the findings of Lath et al. and Alalwani et al [13,19]. However, few studies found C3 to be the most common immune deposit [2-5,22]. In line with previous studies, IgA was commonly noted when the biopsy was done from lesions of < 72 hours duration and C3 was found in lesions more than 72 hours [11,22]. In our study, one patient demonstrated a vascular full-house pattern on DIF showing deposition of all three immunoglobulins and C3 with 3+ intensity. In the study by Lath et al., a full-house vascular pattern of DIF deposit (IgA, IgM, IgG, C3) was seen in 4 cases with deposition along the dermo-epidermal junction in 3 cases which is a feature of lupus vasculitis [13]. In our study, deposition along the dermo-epidermal junction was not seen, and the case was not associated with SLE.

**Table 6:** Comparison of direct immunofluorescence (DIF) findings reported in previous studies Table 6: Comparison of direct immunofluorescence (DIF) findings reported in previous studies [2-5,13,22]

Study	Number of study subjects	DIF done	DIF positive	IgA positivity	IgM positivity	IgG positivity	C3 positivity
Index study	61	56/61 (91.8%)	36/56 (64.2%)	28/56 (50.0%)	7/56 (12.5%)	3/56 (5.4%)	18/56 (32.1%)
Grunwald et al. [22]	40	40 (100%)	37/40 (92%)	17%	25%	28%	40%
Sais et al. [3]	160	102/160 (63.8%)	84.3%	64.7%	49.0%	42.2%	80.4%
Bouiller et al. [4]	112	99 (88.4%)	84/99 (84.8%)	34/99 (34.3%)	36/99 (36.6%)	11/99 (11.1%)	77/99 (77.8%)
Lath et al. [13]	430	198/430 (46.04%)	119/198 (60%)	70/198 (35.4%)	49/198 (24.7%)	37/198 (18.6%)	60/198 (30.3%)
Gupta et al. [2]	50	23/50 (46%)	17/23 (73.9%)	10/23 (43.5%)	12/23 (52.2%)	6/23 (27.1%)	12/23 (52.2%)
Khetan et al. [5]	61	40/61 (65.6%)	Not mentioned	11/40 (27.5%)	10/40 (25%)	12/40 (30%)	19/40 (47.5%)

Our study showed a significant association between DIF positivity and palpable purpura and cutaneous necrosis; whereas, the urticated lesions were associated with a negative DIF (P-value<0.05). So, it is preferable to do a biopsy from a palpable purpuric lesion rather than from nonpalpable purpuric macule and urticarial papules and plaques.

 A recent study reported extracutaneous manifestation in 33.3% of cases of cutaneous vasculitis. Around 70% (46 out of 66 cases) of those who had extracutaneous manifestations had DIF positivity. The authors concluded that to avoid indiscriminate use of the DIF test, the presence of extracutaneous manifestations can reliably guide case selection for undergoing DIF in cutaneous vasculitis [21]. Previous studies have found significantly more renal, gastrointestinal, and musculoskeletal systems involvement in cases with positive vascular IgA deposit [19]. We found a significant association between vascular IgA deposit on DIF with gastrointestinal involvement (P-value= 0.039). Thus, an underlying gastrointestinal involvement should be suspected and investigated in patients with IgA deposition on DIF, even if not clinically evident. The association between systemic involvement and other DIF findings was not significant in our study.

Sais et al. showed that the positivity of the DIF was inversely correlated with the age of the biopsied lesion (P-value<0.001) [3]. Our finding did not corroborate this observation, as most of the biopsies were done from the early lesions (<48 hours old) for the DIF study. Of the 45 cases in whom DIF was done, in 32 cases, a biopsy was done before 48 hours of onset, whereas in 13 cases, lesions were biopsied after 48 hours. We did not find a significant association between the DIF findings and our patients’ clinical and histopathological severity.

Few studies correlating the severity of vasculitis-induced kidney injury and plasma levels of C3c and C4 in patients with ANCA-associated vasculitis have been attempted in recent years. Our study was novel as, despite an extensive literature search, we could not find any previous studies that attempted to correlate serum C3 and C4 levels with clinical and histopathological severity pertaining to the cutaneous involvement in CSVV [23].

There were a few limitations in the study. The sample size was small. The results of our study may not be generalized as it was conducted in a single tertiary care center in India. Recall bias may have contributed while correlating the duration of illness and the skin lesions with the histopathology features, complement levels, and DIF findings.

## Conclusions

In conclusion, serum C3 and C4 levels did not correlate with clinical and histopathological severity in cases of CSVV and are not predictors of disease severity. Similarly, DIF did not correlate with clinico-histopathological severity and may not indicate CSVV disease activity. Clinically, the presence of palpable purpura and cutaneous necrosis is associated with positive DIF. A positive IgA deposition on DIF will indicate gastrointestinal involvement. However, a multicentric study on a larger sample will help in confirming our findings.
